# Beneficial Effects of Quadri Herb (QH) Ointment on Second‐Degree Burn Scald: An In Vivo Study

**DOI:** 10.1155/bmri/4198960

**Published:** 2026-06-04

**Authors:** Bardia Zamani Ranjbar Garmroodi, Milad Bideh, Farahnaz Ramezan, Atefeh Ebrahimi, Ebtesam Amirizad, Atefeh Nasiri, Roghayeh Sharafi, Masoumeh Barik lou, Masoumeh Shafiei, Mahdieh Rajabi Moghadam, Niloofar Honari, Asghar Zarban

**Affiliations:** ^1^ Department of Clinical Biochemistry, Birjand University of Medical Sciences, Birjand, South Khorasan Province, Iran, bums.ac.ir; ^2^ Research and Development (R & D) Section, Hayyan Tech Pharmed Co, BuAli (Avicenna) Research Institute, Mashhad University of Medical Sciences, Mashhad, Razavi Khorasan Province, Iran, mums.ac.ir; ^3^ Department of Genetics, Islamic Azad University, Damghan Branch, Damghan, Semnan Province, Iran, mau.ac.ir; ^4^ Molecular Medicine Research Center, Hormozgan Health Institute, Hormozgan University of Medical Sciences, Bandar Abbas, Hormozgan Province, Iran, hums.ac.ir; ^5^ Department of Biochemistry and Biophysics, Medical Sciences Branch, Islamic Azad University, Tehran, Iran, azad.ac.ir; ^6^ Department of Advanced Technologies, Medical Sciences Branch, Islamic Azad University, Tehran, Iran, azad.ac.ir; ^7^ Department of Plant Biotechnology, National Institute of Genetic Engineering and Biotechnology (NIGEB), Tehran, Iran, nigeb.ac.ir; ^8^ Department of Clinical Pathology, Birjand University of Medical Sciences, Birjand, South Khorasan Province, Iran, bums.ac.ir; ^9^ Department of Hematology, Birjand University of Medical Sciences, Birjand, South Khorasan Province, Iran, bums.ac.ir

**Keywords:** *Aloe vera*, *Artemisia aucheri*, burn-wound healing, *Falcaria vulgaris*, *Scrophularia striata*

## Abstract

**Background:**

Burn injuries are known as one of the main reasons for hospitalizations across the globe. Scalds, which are considered as wet burns, are more common. Many procedures are used to manage the wound healing process; using silver sulphadiazine 1% is one of those routine ways. However, nowadays, traditional medicine findings, along with modern medications, can cooperate with each other to lead to perfect wound care. Thus, the usage of medicinal plants is suggested for this purpose; so, the aim of the present study is to investigate the beneficial effects of a quadri herb (QH) ointment based on of medicinal plants: *Scrophularia striata*, *Artemisia aucheri*, *Falcaria vulgaris*, and *Aloe vera* on second‐degree burn scald.

**Methods:**

A total of 42 male Wistar rats were grouped into 7; after induction of burn scald by the use of hot water, treated groups received QH ointment in concentrations of 0.5%, 1%, 2%, and 5% with respect to herbal extracts; the wound healing process was monitored for 16 days and the results were statistically analyzed. On the final day of intervention, rats were sacrificed, and skin biopsies were used for histopathological evaluations.

**Results:**

Significant reduction of wound percentage (*p* ≤ 0.05) among 2% and 5% groups showed that QH ointment performed better in wound healing compared with the control and positive control groups. Histological analysis also revealed significant increases in granulation and vascular‐fibroblast density with 5% of QH ointment (*p* ≤ 0.01).

**Conclusion:**

It concludes that QH ointment has remarkable effects in the healing of second‐degree burn scalds.

## 1. Introduction

Burn injuries are a prevalent type of trauma that is commonly seen in hospitals worldwide [1]. In this case, there are several types of burn wounds; “scalds,” which are burn wounds caused by wet sources, are particularly common in children and the elderly [1, 2]. These types of burns can be classified into four degrees depending on the depth of the wound and which part of the dermis is affected [2, 3]. Regardless of the source of injury, the healing of burn wounds like any other wounds includes three major phases: (1) inflammatory (reactive), (2) proliferative (reparative), and (3) maturation (remodeling) [2]. Topical treatments are often used to aid in the healing process by reducing local inflammation and preventing infections [4]. However, thermal wounds can also generate reactive oxygen species (ROS), which can lead to lipid peroxidation and local inflammation. Medications for wound healing are mainly focused on the first phase of healing and reducing wound duration [5–7]. Routine order to manage this situation is the usage of medications such as silver sulphadiazine (SSD 1%) topical ointment; despite the beneficial effects of SSD, there are some disadvantages like delayed wound healing, remaining scars, and the possibility of silver adsorption, which may lead to renal toxicity and leucopenia in long term applications [8–10]. A significant number of individuals worldwide rely on traditional remedies to treat skin ailments, with burn‐related conditions accounting for approximately one‐third of cases. Iranian traditional medicine (ITM) has a long history of utilizing plant‐based treatments to address a range of illnesses and pathological states including skin injuries [11, 12].

In this way, *Scrophularia striata*, *Artemisia aucheri*, *Falcaria vulgaris*, and *Aloe vera* are known as those plants that have significant effects on wound healing process; herein, these plants will be introduced briefly.

*S. striata*: It belongs to the Scrophulariaceae family and holds great importance in traditional medicine especially in western Iran [13]; wound healing, control of infectious diseases, and ameliorating inflammations are known as common applications of *S. striata.* It is believed that the effectiveness of *S. striata* in wound healing is attributed to its ability to regenerate fibroblasts, reduce inflammation, and act as an antioxidant, which is linked to its phytochemical composition, including flavonoids, alkaloids, and resin glycosides, among others [14–17].

*A. aucheri*: Belonging to the Asteraceae family, *A. aucheri* is a plant with over 13,000 species worldwide [18]. ITM has found numerous applications for this plant, including wound healing, anti‐inflammatory, antioxidant, antiviral, antimicrobial, and anticancer properties [19, 20]; studies have shown that *A. Aucheri* possesses significant antimicrobial activity against wound infection‐causing bacteria such as *Escherichia coli*, *Staphylococcus aureus*, and *Listeria monocytogenes*. Additionally, its antioxidant compounds aid in wound healing by reducing inflammation at the site of the injury [21–23].

*F. vulgaris*: A plant that belongs to the Apiaceae family, which is found in western Iran provinces [24]. It contains high levels of proteins, starch, resins, amino acids, alkaloids, and fatty acids such as palmitic, stearic, oleic, and linoleic acids. Like others, ITM has identified some specific medicinal applications for this plant, including wound healing, renal clearing, treating gastrointestinal ulcers, and so on [25]. Recent studies have found that *F. vulgaris* has the potential to enhance wound healing and prevent the spread of leishmaniosis‐related infections [26–28]. Additionally, this plant has been shown to promote collagen synthesis and fibroblast proliferation in skin wounds and gastrointestinal ulcers [29, 30].

*A. vera*: It is one of the 400 species of aloe that originates from South Africa and belongs to the Liliaceae family. *A. vera* has been used for a wide variety of medicinal purposes for thousands of years in several cultures [31, 32]. Recent studies have investigated the therapeutic properties of *A. vera*, including its antibacterial, antiviral, anticancer, antioxidant, anti‐inflammatory, skin protective, wound healing, and blood glucose and cholesterol regulating effects. *A. vera* also contains approximately 75 potentially active compounds. Topical and oral administration of *A. vera* stimulates fibroblast activity and proliferation, which significantly increases collagen synthesis due to the presence of glucomannan‐rich polysaccharides and gibberellin, a growth hormone that interacts with fibroblast growth factor receptors, thereby stimulating their activity [33–35].

Based on the previous explanations, the aim of this study is to investigate the wound‐healing properties of the quadri herb, a topical ointment containing extracts of *S. striata, A. aucheri, F. vulgaris,* and *A. vera* on second‐degree burn scalds in vivo.

## 2. Material and Methods

### 2.1. Materials

The following materials were used in the study; each plant was prepared freshly and purchased from local sellers from different parts of the country of Iran: *S. striata* and *A. Aucheri* from the Lorestan province, *F. vulgaris* from the Kermanshah province, and *A. vera* from the South Khorasan province. Further validations about the originality of plants were applied by presenting them to the official herbarium center (Mashhad Research Center for Plant Sciences, Mashhad, Iran) and approved via dedicating voucher‐specific numbers presented in Table 1. For the extraction operations, 99.9% ethanol was purchased from Merck Company (Merck Co, Germany), and double‐distilled water was prepared instrumentally using a water purification instrument. Eucerin, as the ointment basis, was also purchased from the Orand company (Orand Co, Isfahan, Iran).

**Table 1:** Voucher‐specific numbers for plants.

**Plant**	**Family**	**Voucher sp. No**
*Artemisia aucheri* Boiss	Asteraceae	E1176‐FUMH
*Aloe vera* (L.) Burm. f.	Asphodelaceae	E1177‐FUMH
*Falcaria vulgaris* Boiss	Apiaceae	E1178‐FUMH
*Scrophularia striata* Boiss	Scrophulariaceae	E1179‐FUMH

### 2.2. Herbal Extraction

Crude extracts of plants were used in the research; before any operations, plants were washed, dried in an oven (37°C), and pulverized using a grinder. Then the following procedure was applied for each one.

#### 2.2.1. *S*. *striata*

*S. striata* Boiss crude extract was prepared using Tanideh et al. [36] method with slight modifications; according to the procedure, 100 g of dried herb soaked at 400 ml of 70% ethanol as the solvent and placed in the dark for 3 days under constant shaking. After 3 days, the solution was filtered using a paper filter (Whatman, Germany) and then the solvent was evaporated using a vacuum evaporator (Heidolph, Germany); the remaining aqueous solvent was also eliminated by freeze dryer (FD‐5010‐BT, Dena Vacuum Co, Iran) apparatus.

#### 2.2.2. *A*. *aucheri*

*A. aucheri* Boiss crude extract was prepared using Allahtavakoli′s [37] method with slight modifications; according to the procedure, 50 g of dried herb was soaked at 500 ml of solvent with the ratio of 1:1 from double‐distilled water and ethanol; then the solution was placed in the dark into the water bath with an approximate temperature of 45°C for 2 days. After 2 days, the solution was filtered using a paper filter (Whatman, Germany), and then the solvent was evaporated using a vacuum evaporator (Heidolph, Germany), the remaining aqueous solvent was also eliminated by freeze dryer apparatus.

#### 2.2.3. *F*. *vulgaris*

Crude ethanolic extract of *F. vulgaris* Boiss was prepared according to Zangeneh et al. [38] protocol with some modifications; according to the procedure, 100 g of dried herb soaked at 450 ml of 99.9% ethanol as the solvent and placed in the dark for 2 days under constant shaking. After 2 days, the solution was filtered using a paper filter (Whatman, Germany), and then the solvent was evaporated using a vacuum evaporator (Heidolph, Germany).

#### 2.2.4. *A*. *vera*

*A. vera* (L.) Burm.f. gel extract was prepared using Farzadinia et al. [39] protocol; according to this method, herbal leaves were chopped and the gel extracted from them. Immediately after that, the gel was transferred into the freeze dryer apparatus; the final white powder then was stored at −20°C in the dark.

### 2.3. Measurement of Phenolic Content and Antioxidant Activity of Extracts

The total phenolic content (TPC) of the extracts was measured by the method of Folin–Ciocalteu reagent (FCR); the antioxidant activity of extracts was evaluated by diphenyl‐1‐picrylhydrazyl (DPPH) radical scavenging assay (RSA); each mentioned test was performed using commercial kits of Zantox (Kavosh Ariyan Azma Co, Iran).

### 2.4. Quadri Herb: Ointment Preparation

To synthesize the ointment, first, a stock of each extract based on previous studies was prepared. In this way, for *S. striata* a 10% (w/v) stock, for *A. aucheri* a 0.01% (w/v) stock, for *F. vulgaris* a 5% (w/v) stock, and for *A. vera* a 2% (w/v) stock, all except *A. vera* in hydroalcoholic solvent (Ethanol 1:1 H_2_O) were prepared. Then, a certain amount of Eucerin, depending on the treatment, was weighed and placed on a hotplate at 50°C under constant stirring. After homogenization of Eucerin, the temperature was brought below 40°C, and then the extracts were added depending on the concentration of the ointment under continuous stirring. The prepared ointments at concentrations of 0.5%, 1%, 2%, and 5% were freshly used for the treatment.

### 2.5. Animals

A total of 42 male Wistar rats with a mean weight of 250 ± 50 g were used in the study; the animals were grouped by seven and kept in large standard poly ester cages in the Experimental Research Center of Birjand University of Medical Sciences under the artificial circumstances of a 12‐hour dark/light cycle and a temperature of 22 ± 2^°^C; during the intervention, the animals were fed with nutritional pellets and plumbing water; the classifications of the treatment are also shown in Table 2.

**Table 2:** Group classification in the study.

**Group (*n* = 6)**	**Group number**	**Treatment**
Control	1	NO Treatment
Negative control (NC)	2	Eucerin (topical base ointment)
Positive control (PC)	3	Silver sulphadiazine (SSD) 1%
Treatment	4	0.5% Ointment (quadro herb)
	5	1% Ointment (quadri herb)
	6	2% Ointment (quadri herb)
	7	5% Ointment (quadri herb)

### 2.6. Study Design and Burn Induction Model

**Figure 1:**
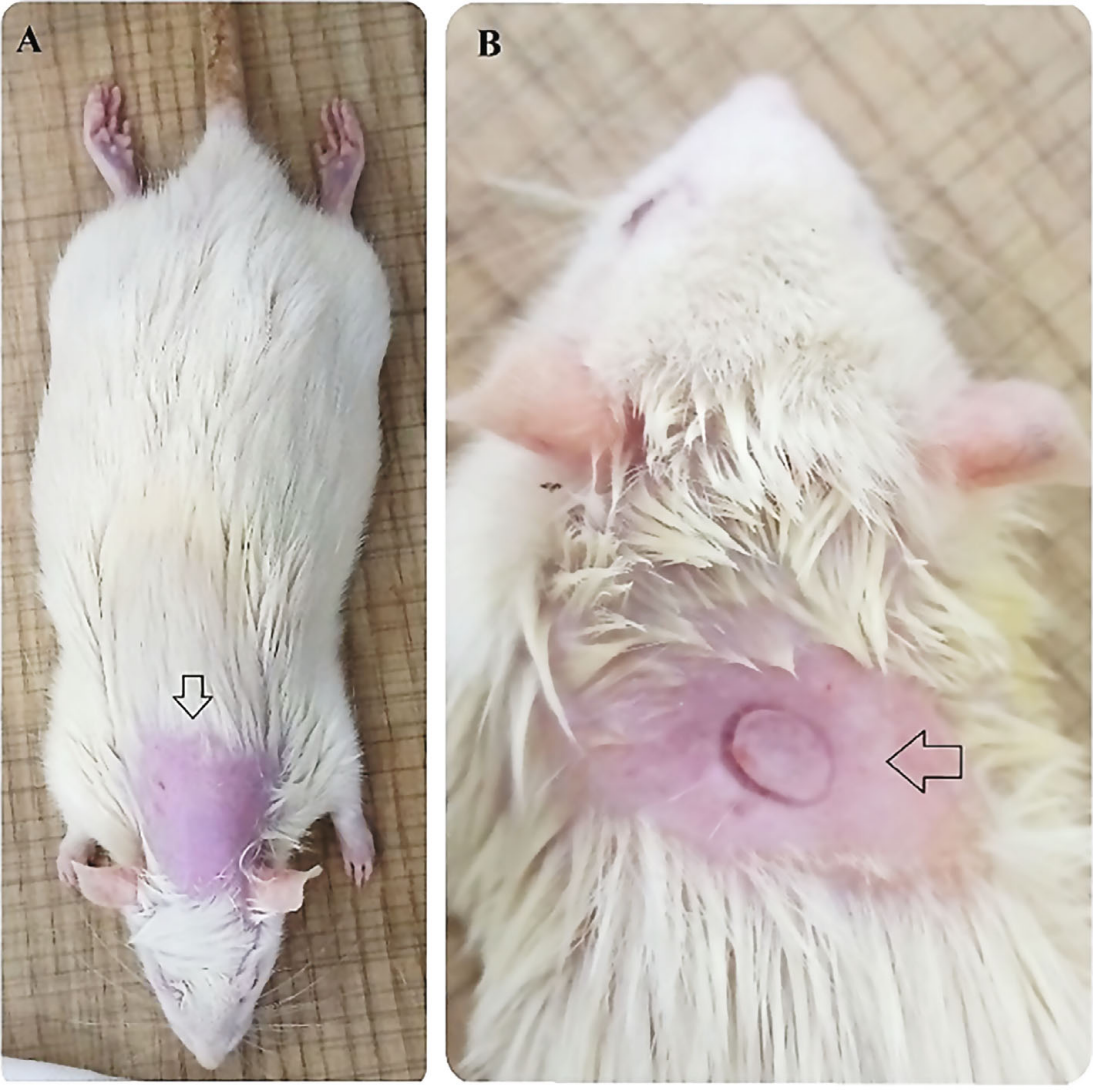
Burn induction in male Wistar rats using standardized scalding burn model. (A) An anesthetized animal shaved and prepared to be burned and (B) induction area (cervical vertebrae located at the dorsal region, hardly accessible for the animal).

The standardized scalding burn model in this research was performed using boiling water [40]; according to the method, a hot water bath with an approximate temperature of 96°C was prepared, and a small glass tube with an external diameter of 8 mm was placed inside. To avoid any disturbance, double‐distilled water was used in the bath. Then, for preparing the animals to induce burn, they were anesthetized via a ketamine‐xylazine mixture intraperitoneally, and the hair on the dorsum region was shaved off to ensure an even burn to the wound. The dorsum is considered as an ideal choice because it is difficult for the animal to reach there and disrupt the intervention; after the preparation process, the tube that contains hot water (≥ 95°C) was put in the dorsum area and held for about 45 s (Figure 1). Now the burn has been induced and the wound area is washed out with normal saline solution. Note that this day is considered as Day 0.

### 2.7. Treatment and Termination of Intervention

The standardized topical treatment regimen was administered daily to the wound site over a period of 16 consecutive days. This application was conducted without the use of occlusive dressings, allowing the ointment‐covered wound to maintain exposure to ambient air. This approach could possibly facilitate optimal wound healing and enhance the efficacy of the ointment under aerobic conditions and also prevent animals from scratching the dressing or any interference. Upon completion of the predetermined study timeline, the intervention was deemed to have ended. Given that the subjects (rats) were no longer suitable for further scientific investigations, the decision was made to humanely terminate them in accordance with established ethical protocols via the “euthanasia” method. Thus, euthanasia was performed using a CO_2_ chamber by a competent person who was certified by the Ethical Committee of Birjand University of Medical Sciences. It is noteworthy that animal termination via a CO2 chamber is recognized as a humane, painless, stress‐free, and ethically sanctioned method within the scientific community and follows the guideline [41, 42].

### 2.8. Measurement of Wound Healing

The wound surface area was calculated computationally using software Image J (V 0.1.5), by regarding the calculated surface, the wound percentage also is calculable using Equation (1):

(1) Wound Percentage=wound surface at the mentioned daywound surface at the Day 0∗100.

### 2.9. Histopathological Examinations

The skin tissues were dissected and fixed in a 10% neutral buffered formalin solution for 48 h at the end of the experiment. They underwent the standard process of tissue sectioning by cutting at a thickness of about 5 *μ*m using a microtome device (Did Sabz Co in Iran). Hematoxylin and eosin (H&E) staining was applied to the prepared tissue sections, which were then examined for histopathological changes using a light microscope (Olympus, Japan). To assess the histological states, the method by Sultana et al. [43] was applied; in this method, by slight modification, the qualitative scoring system converts to semiquantitative evaluation. Therefore, the basis of scoring is as follows: 1, absent; 2, scanty amount (minimum or a few); 3, moderate amount; and 4, profound (or maximum amount) [44]. Note that four histopathological markers have been investigated among skin biopsies including vascular and fibroblast density, tissue granulation, and inflammation. To assess mentioned markers in skin biopsies, the number of inflammatory cells like leukocytes and fibroblasts were counted using Image J (V.1.5) software and reported as inflammation status and fibroblast density. To evaluate granulation of biopsies, both sides of the epidermis and dermis layers were compared in each group using Image J software. Vascular densities were also observed in each section of a biopsy and finally calculated as mean ± standard error of the mean.

### 2.10. Statistical Analyses

The statistical analysis was carried out using Statistical Package for the Social Sciences (SPSS) software (V.22). Kolmogorov–Smirnov test was used in order to determine the normal distribution of data by considering *p* ≥ 0.05. one way ANOVA along with Tukey test used for normal distributed data; Kruskal–Wallis, followed by Mann–Whitney *U* tests were used to evaluate the significant changes between multiple groups in nonparametric status or anormal distributed data. A *p* ≤ 0.05 has been considered as the significant value. All the results were expressed as mean ± standard error (mean ± SE).

## 3. Results

### 3.1. Measurement of Phenolic Content and Antioxidant Activity

The TPC and antioxidant activity of each extract are shown in Table 3; based on the table, the maximum amount of phenolic content and antioxidant activity belongs to the *S. striata* hydroalcoholic extract.

**Table 3:** Total phenolic content (TPC) and antioxidant capacity (TAC) of crude extracts.

**Sample**	**Total Phenolic Content**	**Total Antioxidant Capacity**
*Scrophularia striata* Boiss	1016.5 ± 14.5	1780 ± 20
*Artemisia aucheri* Boiss	870 ± 22	721.5 ± 3.5
*Falcaria vulgaris* Boiss	494 ± 4.0	965.5 ± 5.5
*Aloe vera* (L.) Burm. f.	610 ± 12	537.5 ± 16.5

*Note:* All values are expressed as mean ± standard error of the mean (S.E).

TPC unit, mg of Gallic acid equivalent/mg of extract.

TAC unit, *μ*M of Trolox/mg of extract.

### 3.2. Wound Healing Monitoring

**Figure 2:**
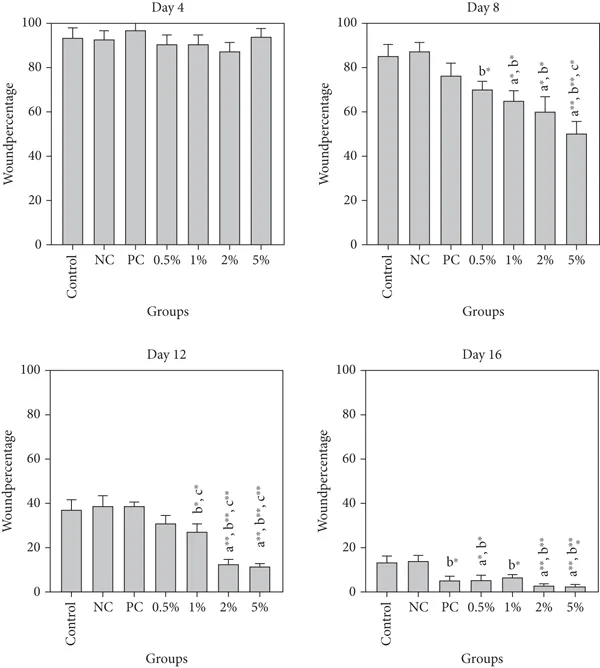
Wound percentage among treated groups at four equal time intervals, a: Compared with control; b: compared with negative control (NC); c: compared with positive control (PC: SSD 1%). ∗*p* value ≤ 0.05; ∗∗*p* value ≤ 0.01; ∗∗∗*p* value ≤ 0.001.

Table 4 indicates the wound healing process during the intervention, whereas Figure 2 exhibits the change in wound percentage among treated groups at four equal time intervals of 4, 8, 12, and 16 days. According to Figure 2, changes in wound percentage between control versus negative and positive control (SSD 1%) groups were insignificant; however, the mean values decreased over time. On the 8th day, significant reductions of wound percentage were observed among the groups treated with QH ointment for the concentrations of 1%, 2% (*p* ≤ 0.05), and particularly for 5% (*p* ≤ 0.01). By the end of the intervention on the 12th and 16th days, it was evident that QH ointment, in all concentrations, had a significantly better performance in wound healing compared with the control and also positive control groups (*p* ≤ 0.05).

**Table 4:** Wound healing process during intervention.

**Time**	**Group**						
	**Control**	**NC**	**PC: SSD 1%**	**0.5% Ointment**	**1% Ointment**	**2% Ointment**	**5% Ointment**
Day 4	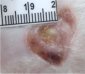	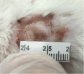	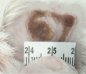	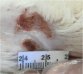	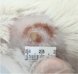	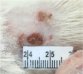	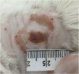
Day 8	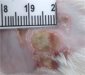	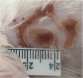	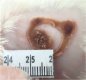	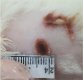	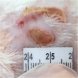	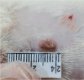	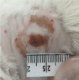
Day 12	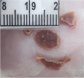	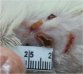	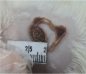	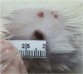	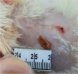	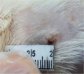	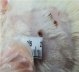
Day 16	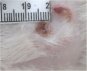	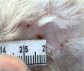	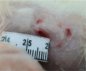	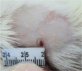	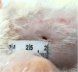	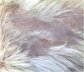	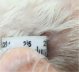

### 3.3. Histopathological Observations

Figure 3 indicates a microscopic panel of burn scalds on the 16th day after intervention; Table 5 also shows microscopical grading for histopathological changes among study groups. As it is clear, all histological features between a normal tissue section and positive control are considered statistically insignificant (*p* > 0.05); QH ointment in the concentration of 5% showed a significant increase in granulation, vascular, and fibroblast density (*p* ≤ 0.01), whereas it has no significant effects on local inflammation (*p* > 0.05) as compared with the control group. QH ointment in the concentration of 2% has a significant increase in granulation (*p* ≤ 0.05) and vascular density (*p* ≤ 0.01), whereas at a concentration of 1% only a significant increase in vascular density (*p* ≤ 0.05) was observed; in these two concentrations, no significant changes in fibroblast density and inflammation were approved (*p* > 0.05). It also sounds that 0.5% QH ointment has a similar performance to SSD 1% in which no significant changes were observed compared with the control group (*p* > 0.05) (Table 5).

Figure 3Microscopic panel of burn scalds on the 16th day of treatment in two microscopic magnitudes: 10× and 40×. (a) Control group, (b) PC (SSD 1%) group, (c) 0.5% ointment groups, (d) 1% ointment group, (e) 2% ointment group, and (f) 5% ointment group. Yellow arrows: keratinocytes; orange arrows: fibroblasts; green arrows: collagen fibers; and red circles: neovascularization region.

**Figure:**
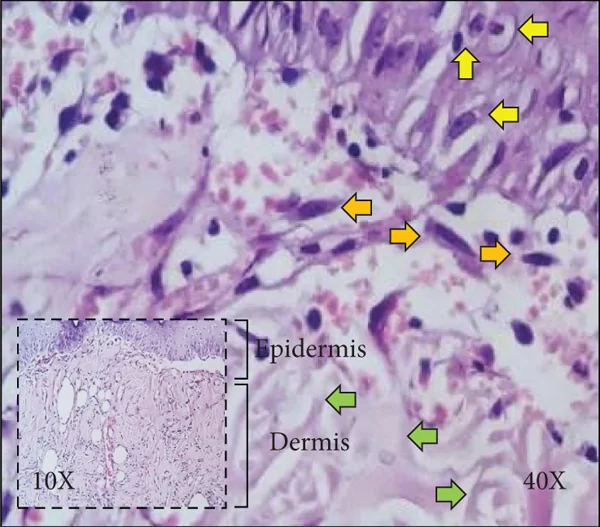
(a)

**Figure:**
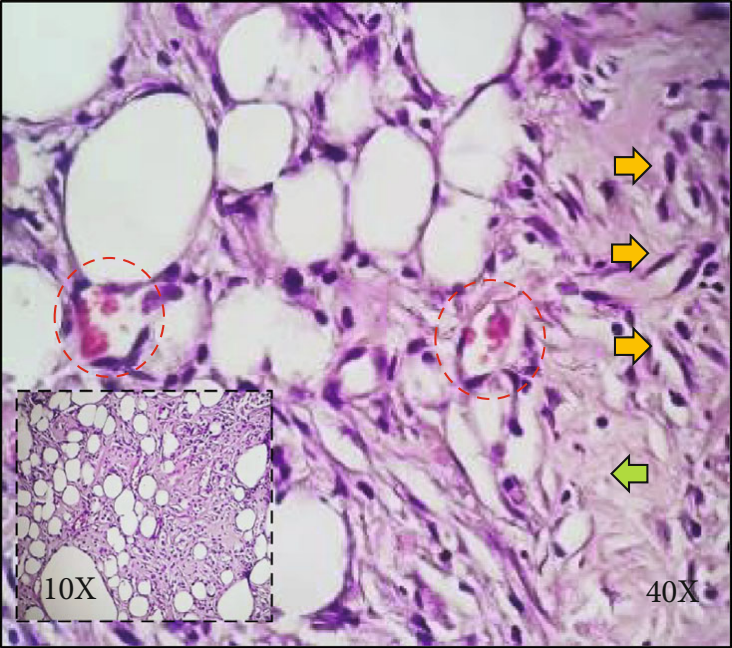
(b)

**Figure:**
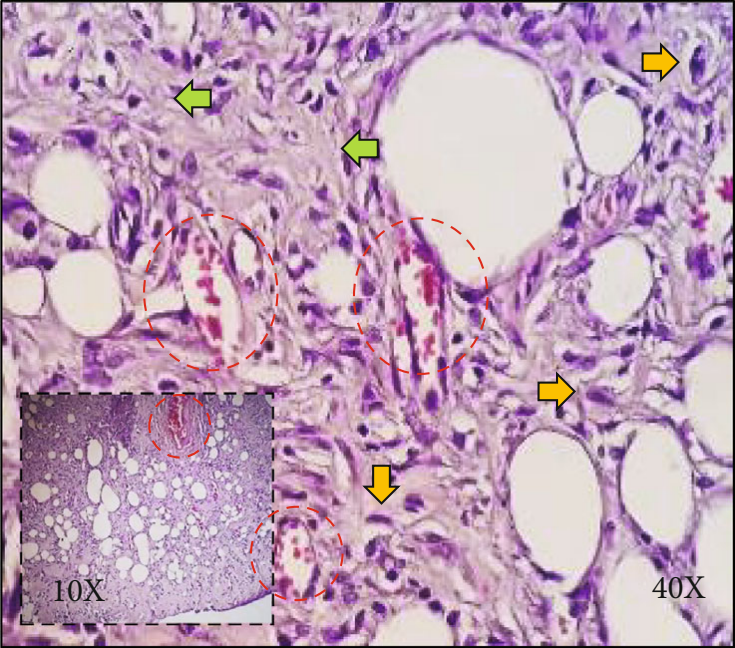
(c)

**Figure:**
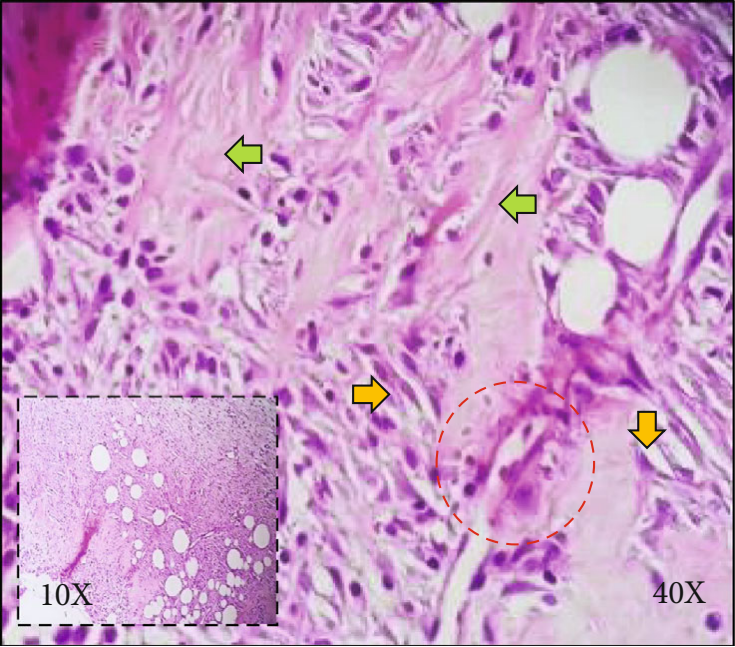
(d)

**Figure:**
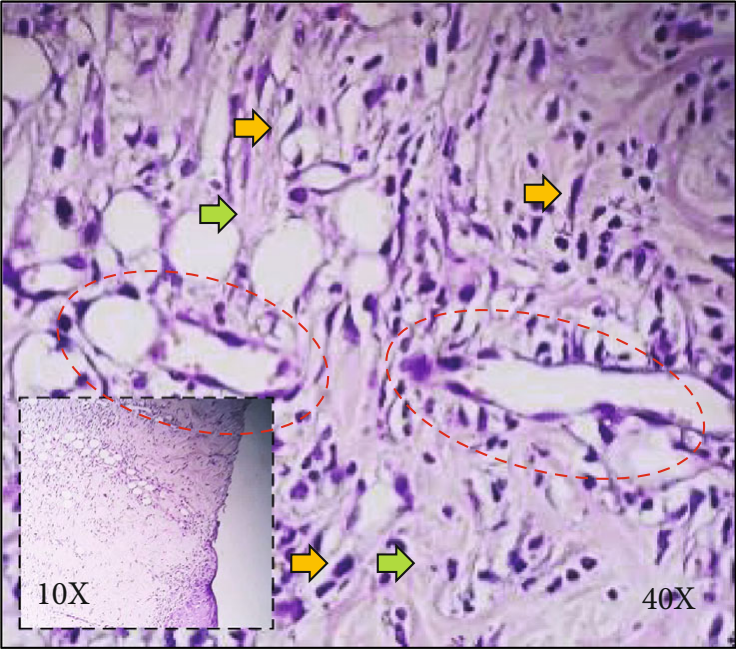
(e)

**Figure:**
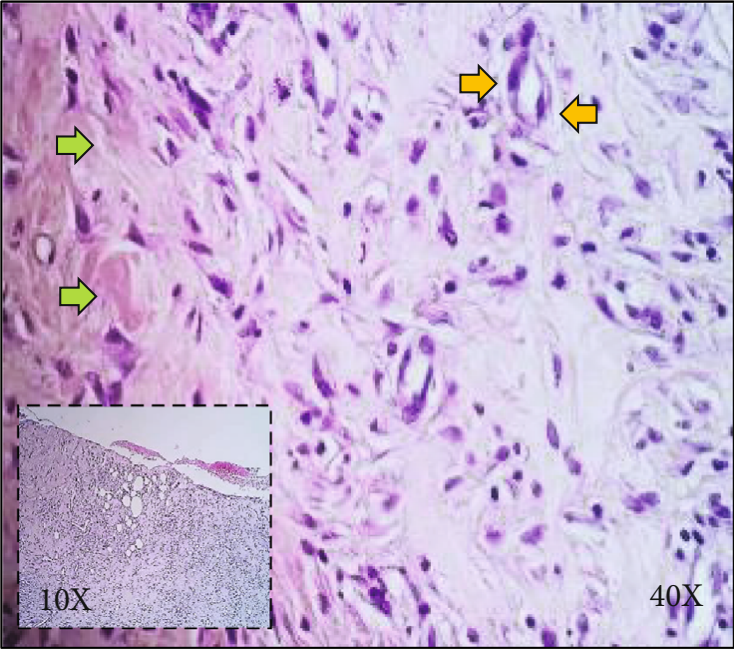
(f)

**Table 5:** Microscopical grading^**#**^ for histopathological changes among study groups.

**Variable**	**Group**					
	**Control**	**PC**	**QH: 0.5%**	**QH: 1%**	**QH: 2%**	**QH: 5%**
Vascular density	1.50 ± 0.22	1.67 ± 0.21	1.83 ± 0.16	2.67 ± 0.21^∗^^a,^ ^∗^^b^	2.17 ± 0.16^∗^^a^	3.83 ± 0.16^∗∗^^a,^ ^∗∗^^b^
Fibroblast density	2.83 ± 0.16	2.67 ± 0.21	2.67 ± 0.21	2.5 ± 0.22	2.5 ± 0.22	3.83 ± 0.16^∗∗^^a,^ ^∗∗^^b^
Granulation	1.67 ± 0.21	1.83 ± 0.16	2.17 ± 0.16	2.33 ± 0.21	2.67 ± 0.21 ^∗^^a,^ ^∗^^b^	3.67 ± 0.21 ^∗^^a,^ ^∗∗^^b^
Inflammation	1.33 ± 0.21	1.33 ± 0.21	1.5 ± 0.22	2.00 ± 0.25	2.00 ± 0.25	1.67 ± 0.21

Abrreviations: QH, Quadri Herb and PC, positive control (SSD 1%).

^a^Compared with control.

^b^Compared with positive control (SSD 1%).

^#^Grading basics: 1, absent; 2, scanty (minimum or a few); 3, moderate; and 4, profound (or maximum amount).

∗*p* value ≤ 0.05; ∗∗*p* value ≤ 0.01; ∗∗∗*p* value ≤ 0.001. Note that all values are expressed as mean ± standard error of the mean (S.E).

## 4. Discussion

Many scientific studies and also traditional medicine, which nowadays is called “complementary medicine,” have claimed that the usage of medicinal plants is one of the best options to manage wound traumas; these plants contain a variety spectrum of natural products such as alkaloids, oils, flavonoids, tannins, saponins, terpenoids, and phenolic compounds, which are responsible for therapeutic applications in the field of wound healing. These phytomedicine compounds are not only safe but also cheap and much easier to use. Despite many studies around the wound healing properties of different plants, the exact mechanism of their action has not been well defined [45–48]. According to the results, it is clear that the herbal base of QH ointment is responsible for its wound healing properties. This ointment contains four herbal extracts that each one has a remarkable antioxidant activity and phenolic content; so, these bioactive compounds give good restorative property to the synthetic ointment. Ointments are usually occlusive shapes of topical formulations; in this way, they stay longer on the surface of skin and trap moisture in to prevent wounds from drying out [49]. This formulation is generally suitable for second‐degree burn scalds; this degree of burn involves both epidermis and dermis. By increasing the depth of the injury, both risks of wound infection and scarring increase. As these scalds are generally painful for patients, wound care is necessary, although surgery is usually not prescribed [1]. It is approved that burn scalds caused by wet sources (≥ 80°C for at least 20 s) could lead to a deep skin injury that involves dermis [50], just like as the applied model in the present study. At this level of injury, the epidermis turns to a dead area and the upper part of the dermis coagulates completely, which is known as the “coagulation zone,” which is easily recognizable from its neighboring zones: stasis and hyperemia [50]. As described earlier, the wound healing process crosses from three or exactly four major steps: (1) coagulation and stasis, (2) inflammation, (3) proliferation/migration/re‐epithelialization/granulation, and (4) remodeling/maturation [51]. At the first moments after burn induction, coagulation and stasis begin; vasodilator molecules and platelets play the main role in this step. Inflammation is the second step. According to wound percentage in time intervals in the present study, it seems that this process lasts for about 8 days after injury; neutrophils, immune cells, and their secretory cytokines are the main characters in this step. These factors initiate the activation of epithelial and fibroblast cells, which play the main roles in the third and fourth steps [51–53]. In the third step, the main goal is to replace dead skin with a new layer. In order to achieve this, mitosis of keratinocytes and fibroblasts is necessary; generation of new blood vessels, which are known as “neovascularization” is essential to supply nutrients and oxygen requirements for this process [51]. As is clear in pathological panels of the present study, fibroblast and vascular density have increased significantly in 1%, 2%, and 5% concentrations of QH ointment, as compared with the normal control group; also, changes in inflammation are considered insignificant, this means that QH ointment could manage both inflammation and re‐epithelialization steps successfully even better than the positive control group (SSD 1%). In about 16 days of the study, the inflammation step passed completely and the wound healing process has come to the final step: granulation. Granulated tissue is transitional in which it is replaced with fibrin/fibronectin matrix about 4 days after injury. In this step, tissue is highly metabolically active, supporting cell proliferation and protein synthesis specifically collagen, which synthesized by wound fibroblasts along with neovascularization turns tissue appearance into pinkish‐red [54]. Obviously, QH ointment at the concentration of 5% could remarkably increase granulation in the wound area.

As it mentioned before, the whole medicinal activity of QH ointment is directly linked to its herbal nature; so, different studies have also claimed that each plant, which was used in the ointment has such wound healing effects. For instance, Haddadi et al. [55] reported that hydroalcoholic extract of *S. striata* could promote wound healing by stimulation of human dermal fibroblasts and endothelial cells proliferation along with enhancement of angiogenesis at 1 mg/mL dosage in vitro. Zarmehri et al. [17] also showed that hydroalcoholic extract of *S. striata* at concentrations of 2.5% and specifically 5% (w/v) can be significantly effective on second‐degree burn wounds in vivo. Tandideh et al. [36] claimed that ethanolic extract of *S. striata* at concentrations of 10% could have beneficial effects in burn wounds that are infected by *Pseudomonas aeruginosa* in vivo. *A. aucheri* also has similar effects on wound healing processes; Allahtavakoli [37] reported that hydroalcoholic extract of *A. aucheri* at a concentration of 100 mg/mL (or 10% w/v) accelerates skin wound healing in vivo. Karimi et al. [56] also proved that aqueous extract of spring‐harvested *A. aucheri* at a concentration of 0.4 mg/mL has a great fatality effect on *Leishmania major* promastigotes lesions in vitro and in vivo. Shakibaie et al. [57] also reported that 5% (w/v) ethanolic extract of *F. vulgaris* could effectively increase wound healing in vivo. According to the results of Goorani et al. [28] study, *F. vulgaris* aqueous extract ointment at a concentration of 3% (w/w) can treat cutaneous wounds in vivo. In another study, Farzadinia et al. [39] claimed that HMF ointment containing *A. vera*, honey, and milk at concentrations of 5% (w/v) could show remarkable anti‐inflammatory and wound healing activities on second‐degree burn wounds in vivo. Chithra et al. [58] also claimed that wounds treated either by topical application or oral administration of *A. vera* could have positive effects on collagen synthesis and thus healing in vivo.

## 5. Conclusion

These findings suggest that QH ointment, containing four medicinal plants of *S. striata*, *A. aucheri*, *F. vulgaris*, *and A. vera* could be more effective than SSD 1% ointment in improving second‐degree burn scalds, especially at concentrations of 2% and 5%. Although the herbal constituents of QH ointment have traditionally been used for wound healing, but further investigation is needed to determine their exact mechanism of action. Therefore, it is recommended to use QH ointment for treating second‐degree burn scalds in practice.

## Ethics Statement

The ethical approval of this research was admitted by the IR.BUMS.REC.1399.486.

## Conflicts of Interest

The authors declare no conflicts of interest.

## Funding

All experimental process of the research are performed in Birjand University of Medical Sciences (Birjand, Iran).

## Data Availability

The data that support the findings of this study are available from the corresponding author upon reasonable request.
